# Data-driven evolutionary game models for the spread of fairness and cooperation in heterogeneous networks

**DOI:** 10.3389/fpsyt.2023.1131769

**Published:** 2023-04-28

**Authors:** Jing-Yi Li, Wen-Hao Wu, Ze-Zheng Li, Wen-Xu Wang, Boyu Zhang

**Affiliations:** ^1^School of Systems Science, Beijing Normal University, Beijing, China; ^2^CSSC Intelligent Innovation Research Institute, Beijing, China; ^3^CSSC System Engineering Research Institute, Beijing, China; ^4^Chinese Institute for Brain Research, Beijing, China; ^5^Laboratory of Mathematics and Complex Systems, Ministry of Education, School of Mathematical Sciences, Beijing Normal University, Beijing, China

**Keywords:** cooperation, fairness, inequality aversion, evolutionary game theory, social network

## Abstract

Unique large-scale cooperation and fairness norms are essential to human society, but the emergence of prosocial behaviors is elusive. The fact that heterogeneous social networks prevail raised a hypothesis that heterogeneous networks facilitate fairness and cooperation. However, the hypothesis has not been validated experimentally, and little is known about the evolutionary psychological basis of cooperation and fairness in human networks. Fortunately, research about oxytocin, a neuropeptide, may provide novel ideas for confirming the hypothesis. Recent oxytocin-modulated network game experiments observed that intranasal administration of oxytocin to a few central individuals significantly increases global fairness and cooperation. Here, based on the experimental phenomena and data, we show a joint effect of social preference and network heterogeneity on promoting prosocial behaviors by building evolutionary game models. In the network ultimatum game and the prisoner’s dilemma game with punishment, inequality aversion can lead to the spread of costly punishment for selfish and unfair behaviors. This effect is initiated by oxytocin, then amplified *via* influential nodes, and finally promotes global cooperation and fairness. In contrast, in the network trust game, oxytocin increases trust and altruism, but these effects are confined locally. These results uncover general oxytocin-initiated mechanisms underpinning fairness and cooperation in human networks.

## 1. Introduction

Humans are self-organized to form a variety of social networks, upon which large-scale cooperative behaviors among genetically unrelated individuals persist (1–3). Human cooperation is crucial to the success of the human species and discriminates them from other species (4–6). To maintain cooperation, a preference for fairness in resource sharing is imperative and becomes a social norm (7–11). Despite significant progress in understanding the incentives of cooperation and fairness in spite of the temptation to be selfish, such as reciprocity and reputation (12–15), the emergence and evolution of cooperation and fairness in structured populations remain puzzling (16, 17).

Many efforts have attempted to interpret the effect of social networks on cooperation and fairness, among which a promising hypothesis is enlightened by a discovery in the field of complex networks (18–22). Much empirical evidence demonstrates that a large number of social and economic networks are heterogeneous, consisting of a small fraction of densely connected central nodes and a majority of sparsely connected peripheral nodes (23–26). It is believed that network heterogeneity plays a key role in cooperation and fairness, and several microscopic mechanisms based on social learning and natural selection have been proposed to explain the network reciprocity on cooperation (27–31). However, previous behavior experiments attempting to verify the network reciprocity hypothesis show negative results, and the influence of heterogeneous networks on prosocial behaviors becomes a debate (32, 33). How cooperation and fairness norms are enforced by social networks remains an outstanding problem.

Recent studies on social and behavioral neuroscience have explored the relationship between human behavior and oxytocin, a hypothalamic neuropeptide that has been associated with trust, fairness expectations, and social value representation (34–37). In particular, a placebo-controlled pharmacological study combining oxytocin and heterogeneous networks has shown that intranasal administration of oxytocin to a few central individuals can enhance global cooperation and fairness, but cannot affect global trust (38). Consequently, we hypothesize that heterogeneous networks indeed play a role in cooperation and fairness, but the effect of network heterogeneity is not prominent unless it is in coordination with individual differences in social preferences. Specifically, the leading effect of those prosocial individuals can be amplified by occupying influential nodes and could further exert a global impact on the whole network. It has been shown that oxytocin as a biological basis of prosocial behaviors accounts for the individual difference in social preference (37, 39, 40). We further hypothesize that individual differences are mainly differences in inequality aversion modulated by oxytocin and analyze the general mechanism underpinning fairness and cooperation in network environments initiated by oxytocin through a data-driven approach. Based on data from three network game experiments about fairness, cooperation, and trust (38), we build three evolutionary game dynamic models and reveal a remarkable joint effect of enhanced inequality aversion and network heterogeneity on global prosocial behaviors.

In the rest of this article, we first introduce the three base games and their network extensions adopted in the behavioral experiments (38): ultimatum game (UG) (41–43), two-stage prisoner’s dilemma game with punishment (tPDG) (44–46) (a costly punishment stage is introduced on the basis of the classic prisoner’s dilemma), and trust game (TG) (47–49). We then analyze strategy evolution in the three games on heterogeneous networks. Considering that individuals are bounded rational, we introduce (disadvantage) inequality aversion to the models (7). Specifically, inequality aversion means that people resist inequitable outcomes, and they are willing to give up some material payoffs to move in the direction of more equitable outcomes. We then construct utility matrices that incorporate both the material payoff and the influence of inequality aversion and analyze the evolution processes by replicator dynamic equations (50, 51). Based on the stability analysis of the dynamical systems and the real data of the experiments (38), the inequality aversion parameters are fitted. Our results show that in the UG and tPDG experiments, oxytocin can increase individual inequality aversion, thereby enhancing altruistic punishment, and this effect can be amplified and spread to the entire network through the network structure. In contrast, the trust enhanced by oxytocin fails to diffuse through the network structure to promote the level of prosociality in the TG network. In summary, our study can effectively explain the phenomenon in the behavioral experiments (38) and confirm that the leading effect caused by inequality aversion can be amplified by occupying influential nodes and further improve the level of cooperation and fairness of the whole network.

## 2. The ultimatum game on heterogeneous networks

### 2.1. Ultimatum game

**Figure 1:**
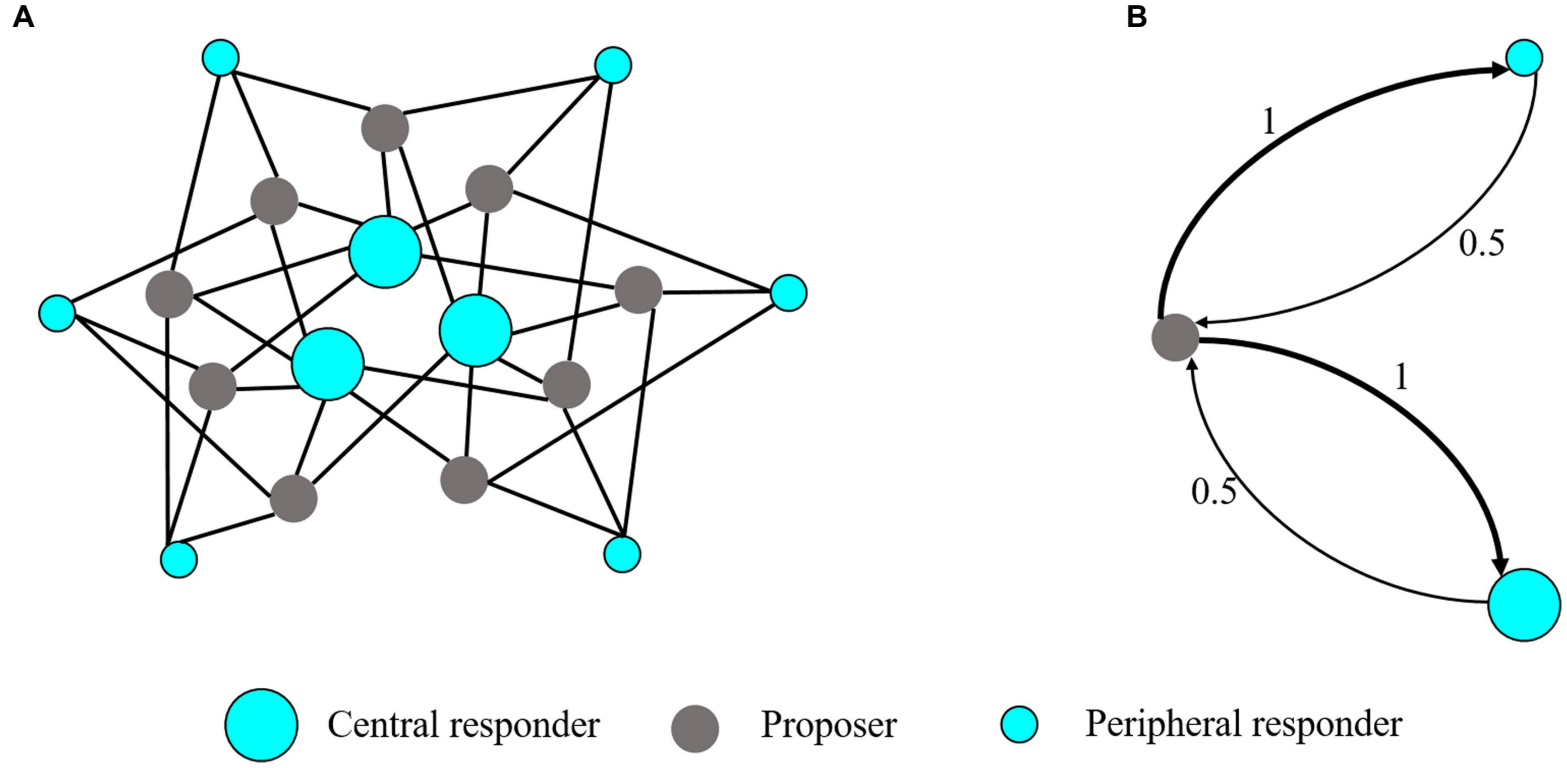
Network structure and mean-field approximation of the ultimatum game. **(A)** The network structure of UG. UG network has three central responders each connected to six proposer neighbors; six peripheral responders, each connected to three proposers and nine proposers each connected to four responders. The payoff of each subject is normalized by his/her number of neighbors. **(B)** The mean-field approximation of the UG network. The simplified system consists of three (types of) nodes, a proposer, a peripheral responder, and a central responder, representing three typical players in the network. The afferent arrows represent interaction with the neighbors of nodes. The thickness of the arrow and the value on the arrow represents the link weight.

The ultimatum game (UG) is a benchmark for studying fairness as a bounded rational behavior (7). Following Han et al. (25), we adopt a minimum acceptance offer (MAO) variant of UG that is simpler for playing on a network, but the essence of UG is not affected. In the networked UG, nine proposers and nine responders are placed at two kinds of nodes in a bipartite network, as shown in Figure 1A. Proposers have an identical neighborhood size with four responder neighbors. In contrast, there are two categories of responders, (i) three central responders with six proposer neighbors and (ii) six peripheral responders with three proposer neighbors. Every proposer connects to two central and two peripheral responders to ensure an unbiased influence from both categories. In each round, a proposer makes a single offer, resource p for each of his/her responder neighbors; a responder claims a single minimum amount, resource q that she/he can accept for all neighbors. Proposers and responders make decisions simultaneously, and every pair of connected subjects shares a fixed amount of resources. For each pair, if p≥q , they make a deal and the proposer gets 1−p and the responder acquires p . If p<q , both get nothing. To control the effect of profit inequality resulting from heterogeneous connections, the actual payoff of subjects in a round is the average over their pairs of games.

### 2.2. Utility matrix

We classify the behaviors of proposers to be two categories: rational (R) with self-interest in payoffs or fairness (F) with fair sharing (52). To simplify our analyses, we assume the polarization of the two categories, i.e., rational proposers offer a small number of resources, s (with s<0.5 ), to responders, and fair proposers offer 50% of resources to responders. Akin to proposers, we classify responders as cooperation (C, acquire any proposals not less than s) and defect (D, reject any proposals). In combination with the influence of disadvantage inequality aversion, we can define the utility of subjects with respect to both payoffs and inequality aversion and construct a utility matrix. Specifically, we assume that the utility of responders resulting from inequality aversion is proportional to the payoff difference, and an internal parameter characterizes the diversity of subjects in responding to inequality (7). The network inhomogeneity accounts for two representative responders: responders occupying central nodes and those occupying peripheral nodes. Thus, we define two utility matrices for the games between proposers and two types of responders (refer to Table 1), where 1−2s is the payoff difference between a rational proposer and a cooperative responder, $\alpha_{1}$ and $\alpha_{2}$ are the internal parameters for central and peripheral responders, respectively, which measure the degree of aversion to unfairness.

**Table 1:** Utility matrix between proposer and responder.

(a) Proposer vs. central responder	C	D
R	$1-s,s-\alpha_{1}\left(1-2s\right)$	0,0
F	0.5,0.5	0,0
(b) Proposer vs. peripheral responder	C	D
R	$1-s,s-\alpha_{2}\left(1-2s\right)$	0,0
F	0.5,0.5	0,0

Our purpose is to estimate the values of internal parameters $\alpha_{1}$ and $\alpha_{2}$ that characterize the influence of oxytocin on the perception of inequality. To accomplish this goal, we employ replicator dynamics to model the evolution of subjects affected by their interactions. To enable analytical results, we reduce the network system with multi-type players based on the mean-field approximation method introduced in Zhang et al. (53) and Pei et al. (54). The basic idea of this method is to approximate the local network structure around a player (i.e., the distribution of different types of players in his/her neighborhood) with the global network structure (which can be derived from the frequencies of different types of edges) and approximate his/her local strategy distributions with the global strategy distributions. We note that this method can be applied to arbitrary networks, but in this article, we only focus on specific networks in Li et al. (38). As shown in Figure 1B, the simplified system consists of three nodes, a proposer, a peripheral responder, and a central responder, representing three typical players in the network. The links in the original network are converted to the interaction weights in the reduced network. The principle of the approximation is as follows:

- Because in the original network, each proposer connects to two central responders and two peripheral responders, in the reduced network the interaction weight from the central responder and the peripheral responder to the proposer is the same.
- Due to the fact that the payoff of each subject from playing with his/her neighbors is normalized by his/her number of neighbors, in the reduced network the sum of incoming link weights should be one.

Based on the approximation principle stemming from local interaction patterns, we can reasonably obtain the reduced network system in Figure 1B.

### 2.3. Replicator dynamics

To analyze the evolutionarily stable strategies for different types of nodes, we formulate replicator dynamics of the reduced network system. We denote the probability of proposers using the R strategy by $\rho_{R}$ , the probability of central responders using the C strategy by $\rho_{C}^{C}$ , and the probability of peripheral responders using the C strategy by $\rho_{C}^{P}$ , respectively. In combination with the utility matrices, we can calculate the expected payoffs of subjects with different roles and strategies as follows:


(1) $$\{\begin{array}{c} E\left(R\right)=\frac{1}{2}\rho_{C}^{C}\left(1-s\right)+\frac{1}{2}\rho_{C}^{P}\left(1-s\right),\\ E\left(F\right)=\frac{1}{4}\rho_{C}^{C}+\frac{1}{4}\rho_{C}^{P},\\ E^{C}\left(C\right)=\rho_{R}\left[s-\alpha_{1}\left(1-2s\right)\right]+\frac{1}{2}\left(1-\rho_{R}\right),\\ E^{C}\left(D\right)=0,\\ E^{P}\left(C\right)=\rho_{R}\left[s-\alpha_{2}\left(1-2s\right)\right]+\frac{1}{2}\left(1-\rho_{R}\right),\\ E^{P}\left(D\right)=0, \end{array}$$

where E(R) and E(F) are the expected payoffs of proposers with R and F strategies, respectively, $E^{C}\left(C\right)$ and $E^{C}\left(D\right)$ are the expected payoffs of central responders with C and D strategies, respectively, and $E^{P}\left(C\right)$ and $E^{P}\left(D\right)$ are the expected payoffs of peripheral responders with C and D strategies, respectively.

Thus, the replicator dynamics for the three types of nodes in the reduced network can be formulated as follows:


(2) $$\begin{array}{c} \{\begin{array}{c} \frac{d\rho_{R}}{dt}=\rho_{R}\left(E\left(R\right)-\bar{E}\right)=\rho_{R}\left(1-\rho_{R}\right)\left(E\left(R\right)-E\left(F\right)\right),\\ \frac{d\rho_{C}^{C}}{dt}=\rho_{C}^{C}\left(E^{C}\left(C\right)-\bar{E^{C}}\right)=\rho_{C}^{C}\left(1-\rho_{C}^{C}\right)\left(E^{C}\left(C\right)-E^{C}\left(D\right)\right),\\ \frac{d\rho_{C}^{P}}{dt}=\rho_{C}^{P}\left(E^{P}\left(C\right)-\bar{E^{P}}\right)=\rho_{C}^{P}\left(1-\rho_{C}^{P}\right)\left(E^{P}\left(C\right)-E^{P}\left(D\right)\right), \end{array} \end{array}$$

where $\bar{E}$ , $\bar{E^{C}}$ , and $\bar{E^{P}}$ are the mean expected payoffs of proposers, central responders, and peripheral responders, respectively.

### 2.4. Stability analysis

The replicator dynamics do not have interior fixed points and have eight boundary fixed points $\left(\rho_{R},\rho_{C}^{C},\rho_{C}^{P}\right)$ , namely, (0, 0, 0), (0, 0, 1), (0, 1, 0), (0, 1, 1), (1, 0, 0), (1, 0, 1), (1, 1, 0), (1, 1, 1), where stable boundary fixed points correspond ESS of the game. We then implement stability analysis for each of the boundary fixed points (see SI for details). In general, Eq. (2) can have multiple stable fixed points. Since R is a dominant strategy for both types of proposers, we are more interested in the stable point with $\left(\rho_{C}^{C},\rho_{C}^{P}\right)=\left(1,1\right)$ . In this case, the only possible stable point is (1, 1, 1), where at this point proposers are rational and responders are cooperative.

Finally, we estimate the values of $\alpha_{1}$ , $\alpha_{2}$ , and s at (1, 1, 1) from the experimental data. From the stability condition, we have


(3) $$\begin{array}{c} \{\begin{array}{c} s<0.5,\\ \frac{\alpha_{1}}{1+2\alpha_{1}}<s,\\ \frac{\alpha_{2}}{1+2\alpha_{2}}<s, \end{array} \end{array}$$

For convenience, let $q_{1}=\frac{\alpha_{1}}{1+2\alpha_{1}}$ and $q_{2}=\frac{\alpha_{2}}{1+2\alpha_{2}}$ . Intuitively, $q_{1}$ (or $q_{2}$ ) represents the acceptance threshold of the central (or peripheral) responders adjacent to the proposer, where offers lower than the threshold will be rejected due to inequality aversion. Thus, we have


(4) $$\begin{array}{c} \{\begin{array}{c} \begin{array}{c} \alpha_{1}=\frac{q_{1}}{1-2q_{1}},\\ \alpha_{2}=\frac{q_{2}}{1-2q_{2}}. \end{array}\\ \; \end{array} \end{array}$$

Eq. (4) implies that the values of the inequality aversion parameters $\alpha_{1}$ and $\alpha_{2}$ can be estimated from the minimum acceptance offers $q_{1}$ and $q_{2}$ . Here, we use data from the UG experiment in the study (38) to fit the parameters. The central nodes were given oxytocin or placebo in the experiment (the settings are the same in the following tPDG and TG experiments). The experimental group (administered oxytocin, OT) and the control group (administered placebo, PL) generated two sets of data, respectively. We use the mean minimum acceptance offers over 60 rounds of the central (or peripheral) responders adjacent to proposers to estimate $q_{1}$ (or $q_{2}$ ; see Table 2). The estimated values of $\alpha_{1}$ and $\alpha_{2}$ for OT and PL groups are shown in Table 3.

**Table 2:** Estimated values of $q_{1}$ and $q_{2}$ in OT and PL groups.

	Central responder ( $q_{1}$ )	Peripheral responder( $q_{2}$ )
OT	0.47	0.49
PL	0.43	0.47

**Table 3:** Estimated values of $\alpha_{1}$ and $\alpha_{2}$ in OT and PL groups.

α	Central responder ( $\alpha_{1}$ )	Peripheral responder( $\alpha_{2}$ )
OT	8.73	19.61
PL	3.17	7.30

**Figure 2:**
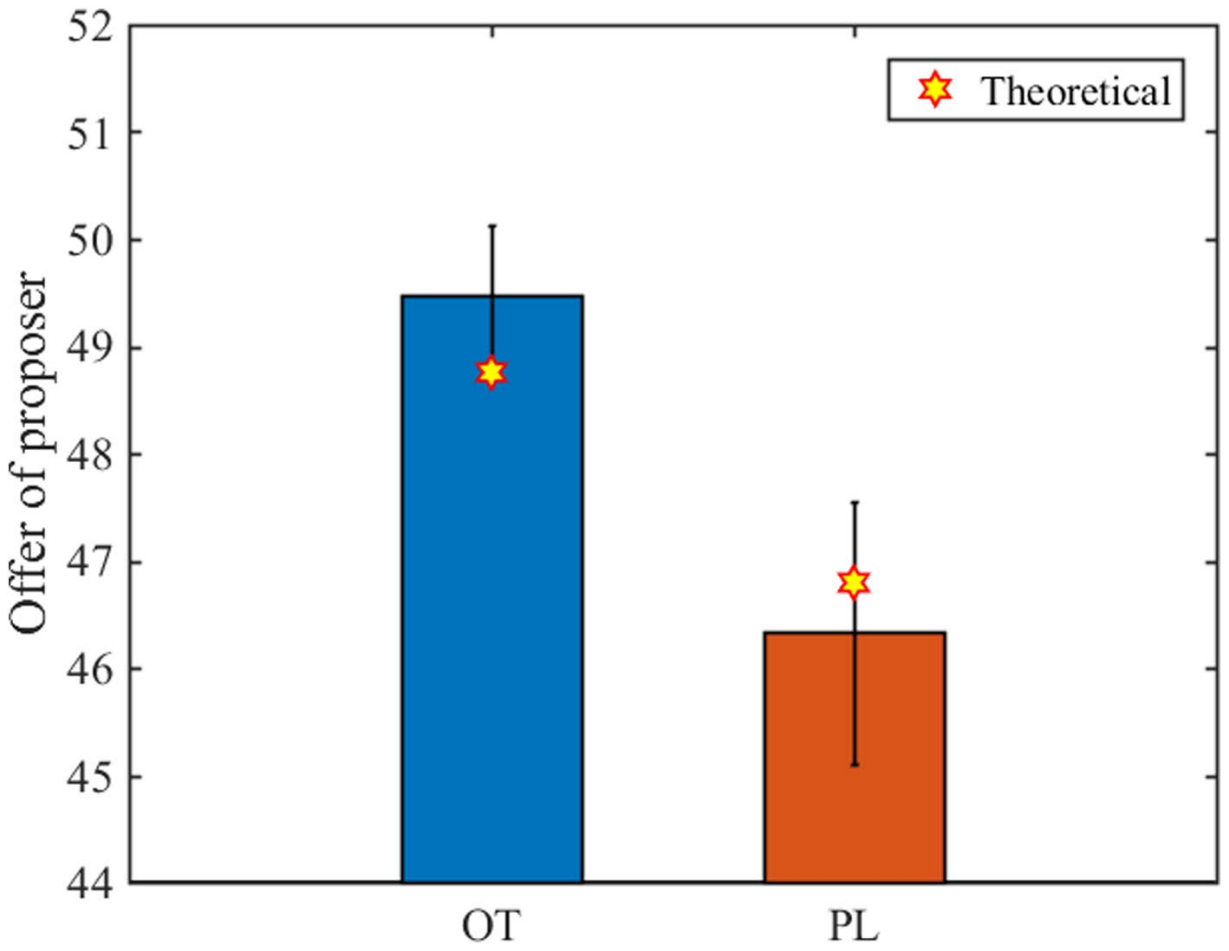
Experimental and predicted results of the proposers’ offer. The blue bar and the red bar represent the mean value (all rounds of data) of the proposers’ offer in the OT group and the PL group, respectively. Each error bar represents the standard deviation, and each yellow six-pointed star represents the theoretical value.

Not surprisingly, $\alpha_{1}$ of the OT group is greater than those of the PL group, which implies that oxytocin indeed promotes inequality aversion. Interestingly, $\alpha_{2}$ of the OT group is also higher. It indicates that oxytocin not only increases the inequality aversion of the central nodes but also spreads this influence to the entire network. Subsequently, we can predict the offer s of proposers determined by $\alpha_{1}$ and $\alpha_{2}$ based on our model. Note that to guarantee a deal with responders, a rational proposer’s offer is confined by the condition $s=\max\left\{q_{1},q_{2}\right\}$ (25). The predicted values of s for both OT and PL groups are shown in Figure 2.

The theoretical predictions are in good agreement with the experiment results, indicating that our model is effective. In particular, our model shows that the inequality aversion of the central responders and peripheral responders can be directly or indirectly increased by oxytocin. This is due to a subtle network effect, inequity aversion of central responders initiated by OT, self-interest of proposers induced by loss aversion, and conditional fairness of peripheral responders, which together constitute a mechanism underpinning the prosocial behaviors. Specifically, due to the endowment effect and loss aversion (55, 56), proposers use the best response strategy to maximize their payoffs and regard the offer to responders as a loss and often match the maximum *q* in their neighbors attempting to make all deals (25). The fact that responders refuse low offers because of inequity aversion resembles costly punishment to proposers. OT stimulates inequity aversion of central responders and imposes more punishment threats to unfair proposers. Despite the insignificant effect of OT exerting on only a small fraction of subjects, the local effect is amplified by central nodes with a larger number of connections. As a result, the central subjects become leaders in driving fairness behaviors *via* costly punishment.

Moreover, two complementary effects nudge network fairness. The first one is the complement among central nodes. Note that each proposer links to two central responders. Thus, only one of the central subjects who is qualified as a leader is sufficient to drive fairness of proposers who attempt to make all deals with their neighbors. The second complementary effect is ascribed to the conditional fairness of peripheral responders who increase their *q* insofar as their proposer neighbors increase their offers. In other words, the responders experience an inner conflict between advocating fairness and loss aversion, and the latter outweighs the former. The leaders help the responders overcome the obstacle of loss aversion and pursue fairness. Conditional fairness as compensation is important to sustain a high level of fairness during evolution in case of the fluctuation of the leader effect occasionally.

Taken together, OT initiates local costly sanctions on unfair behaviors by increasing the inequity aversion of subjects. The local effect is amplified by network heterogeneity and further assisted by conditional fairness and network complementary effects. Finally, the threat of punishment diffuses in the network and a high level of global fairness emerges. The mechanism underpinning network fairness enlightens us to explore network cooperation with costly punishment. We speculate that OT plays a similar role in costly punishment for selfish behaviors, and in combination with subtle network effects, cooperation could be fostered. We next analyze the behavioral evolution of the two-stage prisoner’s dilemma game (tPDG) on the heterogeneous network by building the model to validate our hypothesis.

## 3. The prisoner’s dilemma game with costly punishment on heterogeneous networks

### 3.1. Two-stage Prisoner’s dilemma game

**Figure 3:**
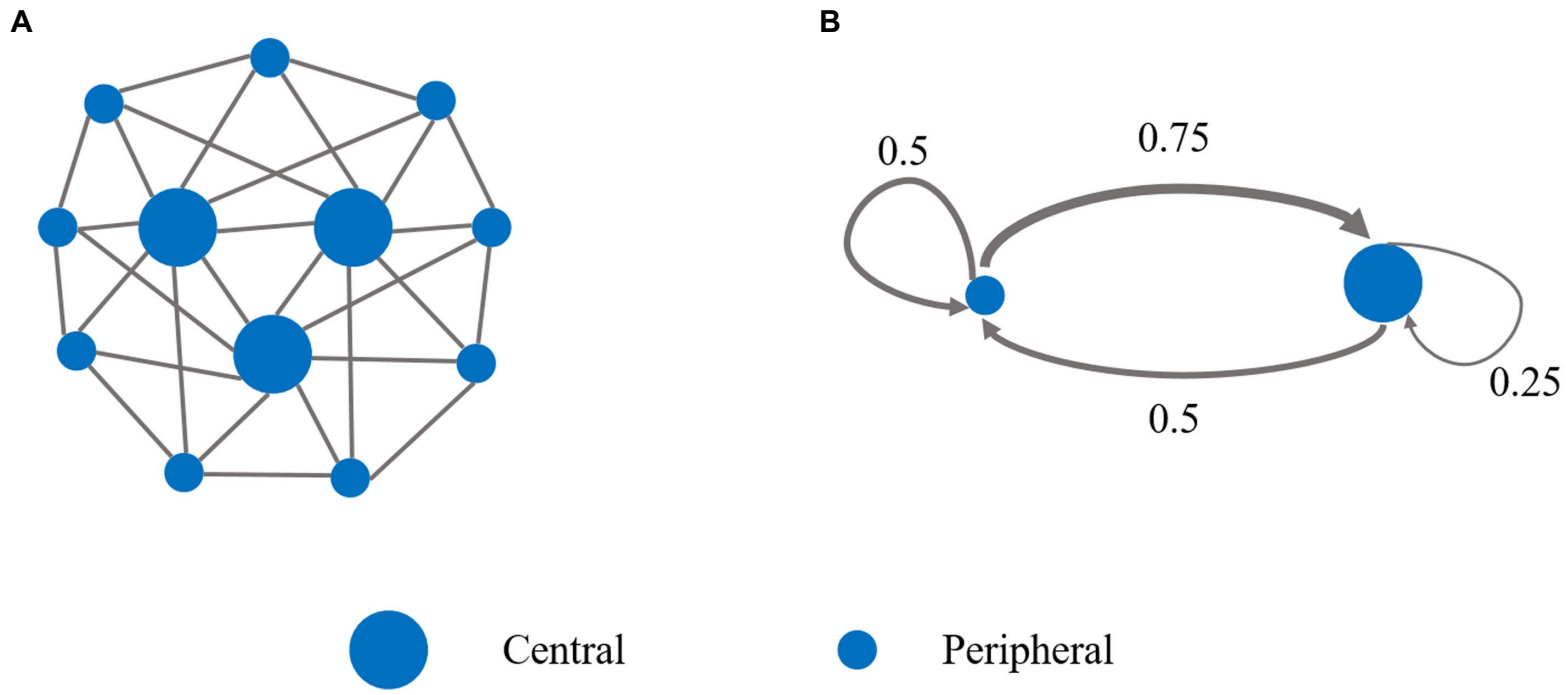
Network structure and mean-field approximation of the two-stage prisoner’s dilemma game. **(A)** There are three central players and nine peripheral players in the tPDG network. Each central player was connected to eight neighbors (two central and six peripheral players), and each peripheral player was connected to only four neighbors (two central and two peripheral players). **(B)** The mean-field approximation of the tPDG network. The simplified system consists of (types of) two nodes, a peripheral node and a central node, representing two typical players in the network. The afferent arrows represent interaction with the neighbors of nodes. The thickness of the arrow and the value on the arrow represents the link weight.

In the network of tPDG, there are two categories of nodes, three central nodes and nine peripheral nodes (Figure 3A), where each central and peripheral node has eight and four neighbors, respectively. To balance the influence of both categories, each peripheral node connects two central and two peripheral nodes, and each central node connects two central and six peripheral nodes. There are two stages in each round. In stage I, subjects choose either cooperate (C) or defect (D), and play with their neighbors simultaneously. The payoffs between each pair of neighboring subjects are calculated according to the payoff matrix (Table 4), where $\hat{T}$ > $\hat{R}$ > $\hat{P}$ > $\hat{S}$ . Similar to UG, the actual payoff of each subject is the average over all pairs of games in a round. In stage II, subjects can opt to costly punish their neighbors choosing D in stage I (57). The cost and punishment are normalized by the neighborhood size.

**Table 4:** Payoff matrix in the prisoner’s dilemma game.

	C	D
C	$\hat{R}$ , $\hat{R}$	$\hat{S}$ , $\hat{T}$
D	$\hat{T}$ , $\hat{S}$	$\hat{P}$ , $\hat{P}$

### 3.2. Utility matrix

To simplify our analyses and modeling processes, we merge the two steps and make an expanded payoff matrix associated with four strategies, i.e., cooperate and not punish (C+N), cooperate and punish (C+P), defect and not punish (D+N), and defect and punish (D+P) (46). The payoff matrix is shown in Table 5, where $\hat{C}$ is the cost of punishing neighbors with D strategy and $\hat{F}$ is the fine of punishment.

**Table 5:** Payoff matrix in the two-stage prisoner’s dilemma game.

	C+N	C+P	D+N	D+P
C+N	$\hat{R}$ , $\hat{R}$	$\hat{R}$ , $\hat{R}$	$\hat{S}$ , $\hat{T}$	$\hat{S}$ , $\hat{T}$
C+P	$\hat{R}$ , $\hat{R}$	$\hat{R}$ , $\hat{R}$	$\hat{S}-\hat{C}$ , $\hat{T}-\hat{F}$	$\hat{S}-\hat{C}$ , $\hat{T}-\hat{F}$
D+N	$\hat{T}$ , $\hat{S}$	$\hat{T}-\hat{F}$ , $\hat{S}-\hat{C}$	$\hat{P}$ , $\hat{P}$	$\hat{P}-\hat{F}$ , $\hat{P}-\hat{C}$
D+P	$\hat{T}$ , $\hat{S}$	$\hat{T}-\hat{F}$ , $\hat{S}-\hat{C}$	$\hat{P}-\hat{C}$ , $\hat{P}-\hat{F}$	$\hat{P}-\hat{C}-\hat{F}$ , $\hat{P}-\hat{C}-\hat{F}$

We speculate that few subjects will employ the D+P strategy. This strategy is not only strictly dominated by D+N but also cognitive dissonant in the sense that defectors punish other defectors. Thus, the payoff matrix can be reduced to three dimensions. Similar to the scenario in the UG [note that an alternative explanation for rejection in UG is that the responder punishes proposers by paying s such that the proposer loses 1-s, see (46)], we assume that the motivation of punishment is inequality aversion, where the willingness to punish defectors is positively related to $\hat{F}-\hat{C}$ (i.e., the efficiency of punishment). Meanwhile, cooperators who are defected may not choose to punish, especially central players tended to exhibit choosing to cooperate without punishing others’ defection in oxytocin (vs. placebo) network (38). We speculate that the underlying reason is a kind of altruism (i.e., maximum group benefit) and may be affected by oxytocin. We further assume that this effect is positively related to $\hat{R}-\hat{P}$ , (i.e., the benefit of mutual cooperation minus mutual defection). Regarding both inequality aversion and dilemma aversion, we have the utility matrix (Table 6) for central subjects, where $\beta_{1}\left(\hat{R}-\hat{P}\right)$ is the increase of utility by avoiding mutual defections, $\beta_{1}$ is an internal parameter capturing the individual difference in dilemma aversion, $\alpha_{1}\left(\hat{F}-\hat{C}\right)$ captures the willingness of punishment because of inequality aversion, and the internal parameter $\alpha_{1}$ measures the degree of inequality aversion that could be affected by oxytocin. For peripheral subjects, we can write a similar utility matrix (Table 6), where $\beta_{2}$ and $\alpha_{2}$ represent the parameters of peripheral subjects.

**Table 6:** Utility matrix in the two-stage prisoner’s dilemma game.

(a) central player	C+N	C+P	D+N
C+N	$\hat{R}$ , $\hat{R}$	$\hat{R}$ , $\hat{R}$	$\hat{S}+\beta_{1}\left(\hat{R}-\hat{P}\right)$ , $\hat{T}$
C+P	$\hat{R}$ , $\hat{R}$	$\hat{R}$ , $\hat{R}$	$\hat{S}-\hat{C}+\alpha_{1}\left(\hat{F}-\hat{C}\right)$ , $\hat{T}-\hat{F}$
D+N	$\hat{T}$ , $\hat{S}+\beta_{1}\left(\hat{R}-\hat{P}\right)$	$\hat{T}-\hat{F}$ , $\;\hat{S}-\hat{C}+\alpha_{1}\left(\hat{F}-\hat{C}\right)$	$\hat{P}$ , $\hat{P}$
(b) peripheral player	C+N	C+P	D+N
C+N	$\hat{R}$ , $\hat{R}$	$\hat{R}$ , $\hat{R}$	$\hat{S}+\beta_{2}\left(\hat{R}-\hat{P}\right)$ , $\hat{T}$
C+P	$\hat{R}$ , $\hat{R}$	$\hat{R}$ , $\hat{R}$	$\hat{S}-\hat{C}+\alpha_{2}\left(\hat{F}-\hat{C}\right)$ , $\hat{T}-\hat{F}$
D+N	$\hat{T}$ , $\hat{S}+\beta_{2}\left(\hat{R}-\hat{P}\right)$	$\hat{T}-\hat{F}$ , $\;\hat{S}-\hat{C}+\alpha_{2}\left(\hat{F}-\hat{C}\right)$	$\hat{P}$ , $\hat{P}$

Our aim is to estimate parameter values and reveal the effect of OT on the internal parameter $\alpha_{1}$ , $\alpha_{2}$ , $\beta_{1}$ , and $\beta_{2}$ . Analog to the case of network UG, we also use mean-field approximation to simplify our analyses. Because of the normalization of payoffs and punishment over neighbors of every subject, the original network can be reduced to a two-node graph with self-loops, as shown in Figure 3B.

In the original graph, a peripheral node connects to two other peripheral nodes and two central nodes, and a central node connects to six peripheral nodes and two other central nodes. Thus, the link weight of the self-loop of the peripheral node is 0.5, the same as the link weight from the central node to the peripheral node. The weight of the self-loop of the central node is 2/(2+6)=0.25 , and the link weight from the peripheral node to the central node is 6/(2+6)=0.75 .

### 3.3. Replicator dynamics

Next, we formulate replicator dynamics equations of the reduced network system. We denote the probabilities of central nodes using C+N, C+P, and D+N strategies by $\rho_{C+N}^{C}$ , $\;\rho_{C+P}^{C}$ , and $\rho_{D+N}^{C}$ , respectively. Similarly, we denote the probabilities of peripheral nodes using C+N, C+P, and D+N strategies by $\rho_{C+N}^{P}$ , $\rho_{C+P}^{P}$ , and $\rho_{D+N}^{P}$ , respectively. In combination with the utility matrices, we can calculate the expected payoffs of subjects with different roles and strategies, see SI for details. The replicator dynamics equations for the two nodes in the reduced network can be formulated as follows:


(5) $$\begin{array}{c} \{\begin{array}{c} \frac{d\rho_{C+N}^{C}}{dt}=\rho_{C+N}^{C}\left[E^{C}\left(C+N\right)-\bar{E^{C}}\right],\\ \frac{d\rho_{C+P}^{C}}{dt}=\rho_{C+P}^{C}\left[E^{C}\left(C+P\right)-\bar{E^{C}}\right],\\ \frac{d\rho_{C+N}^{P}}{dt}=\rho_{C+N}^{P}\left[E^{P}\left(C+N\right)-\bar{E^{P}}\right],\\ \frac{d\rho_{C+P}^{P}}{dt}=\rho_{C+P}^{P}\left[E^{P}\left(C+P\right)-\bar{E^{P}}\right]. \end{array} \end{array}$$

where $E^{C}\left(C+N\right)$ and $E^{C}\left(C+P\right)$ are the expected payoffs of the central node with C+N and C+P strategies, $E^{P}\left(C+N\right)$ and $E^{P}\left(C+P\right)$ are the expected payoffs of the peripheral node with C+N and C+P strategies, and $\bar{E^{C}}$ and $\bar{E^{P}}$ are the mean expected payoffs of the central node and peripheral node, respectively.

We then estimate the values of $\alpha_{1}$ , $\alpha_{2}$ , $\beta_{1}$ , and $\beta_{2}$ by the virtue of experimental results. Note that the replicator dynamics are complicated with a large number of terms. This precludes us from deriving complete stability analyses for Eq. (5). Alternatively, we take the mean proportions of strategies of the last 20 rounds in experiments as the equilibrium points of the replicator dynamics, such that the parameter values in the dynamics can be estimated. Specifically, the (stable) proportions of strategies in OT and PL groups are shown in Table 7 (38). Thus, by inserting the equilibrium points into the replicator dynamics, we can solve the values of $\alpha_{1}$ , $\alpha_{2}$ , $\beta_{1}$ , and $\beta_{2}$ for OT and PL groups, as shown in Table 8.

**Table 7:** Stable proportions of strategies in OT and PL groups.

	OT		PL	
	Central node	Peripheral node	Central node	Peripheral node
C+N	0.18	0.30	0.09	0.15
C+P	0.10	0.09	0.06	0.05
D+N	0.72	0.61	0.85	0.80

**Table 8:** Estimated values of $\alpha_{1}$ , $\alpha_{2}$ , $\beta_{1}$ , and $\beta_{2}$ in OT and PL groups.

	OT	PL
Central node	$\alpha_{1}$ =3.13 $\beta_{1}$ =1.13	$\alpha_{1}$ =3.04 $\beta_{1}$ =1.04
Peripheral node	$\alpha_{2}$ =3.07 $\beta_{2}$ =1.07	$\alpha_{2}$ =3.02 $\beta_{2}$ =1.02

### 3.4. Stability analysis

**Figure 4:**
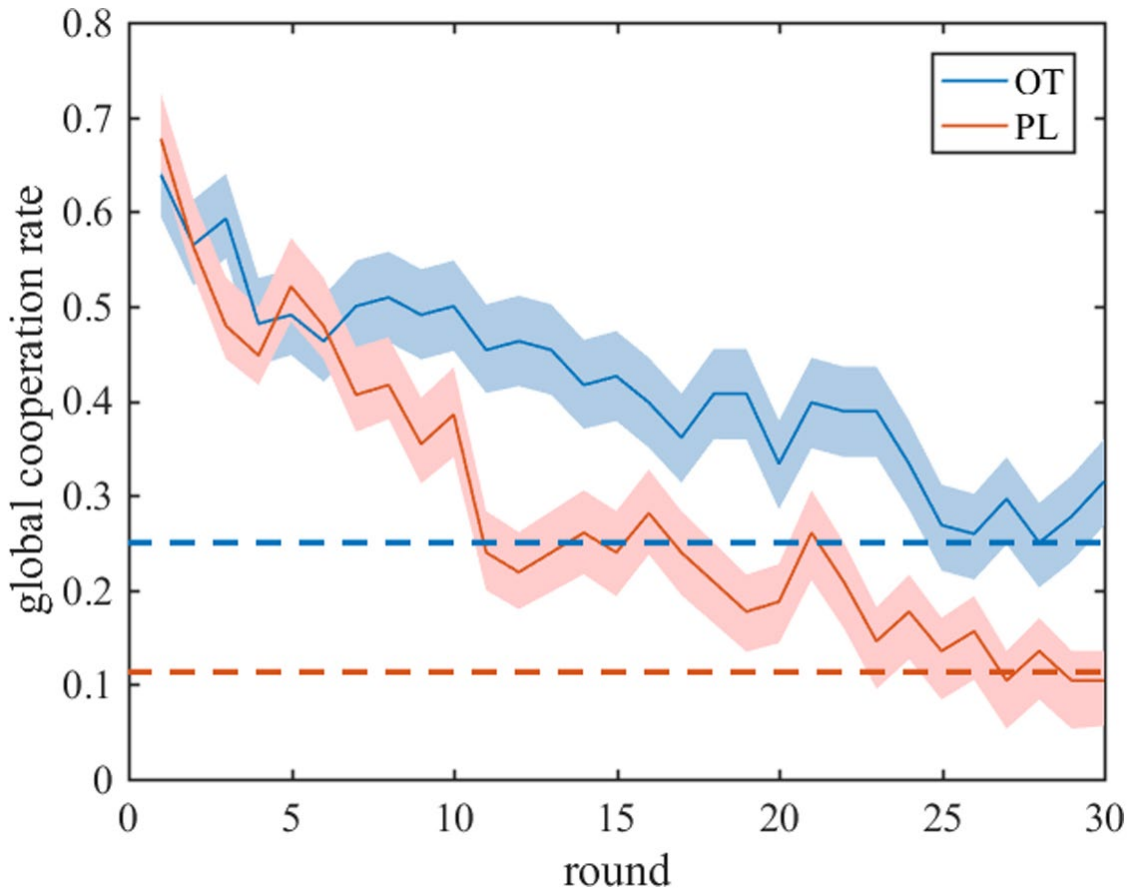
Experimental and predicted results of the global cooperation rate in tPDG. The solid lines represent the time evolution of the global cooperation rate in the iterated tPDG experiments and shaded areas represent standard error. The blue (red) lines represent OT (PL) group. The dotted lines represent the global cooperation rate at the stable fixed point of the replicator equations.

Finally, we implement stability analysis to test if the equilibrium points in the experiments are indeed stable under Eq. (5). We formulate the Jacob matrix and calculated its eigenvalue using the estimated parameter values. We see that the real part corresponding to each eigenvalue of the Jacobi matrices is non-positive (see Supplementary Table S1), which indicates that the equilibrium state in the experiments is stable and can be achieved in our model. Thus, our evolutionary model is valid to model the evolution of cooperative behaviors in the prisoner’s dilemma experiments with costly punishment (see Figure 4).

Our results indicate that oxytocin improves both the inequality aversion parameters $\alpha_{1}$ and $\alpha_{2}$ and the dilemma aversion parameters $\beta_{1}$ , and $\beta_{2}$ . Specifically, the costly punishment in stage II is analogous to rejecting unfair offers in UG, and OT triggers willingness to costly punishment by increasing inequity aversion of central subjects. The local punishment effect is amplified by central nodes and diffuses in the network by virtue of motivating conditional punishment of peripheral subjects. Actually, inspired by the sanction behaviors of central nodes, peripheral subjects’ willingness to costly punishment in OT groups is significantly higher than that in PL groups.

## 4. The trust game on heterogeneous networks

### 4.1. Trust game

**Figure 5:**
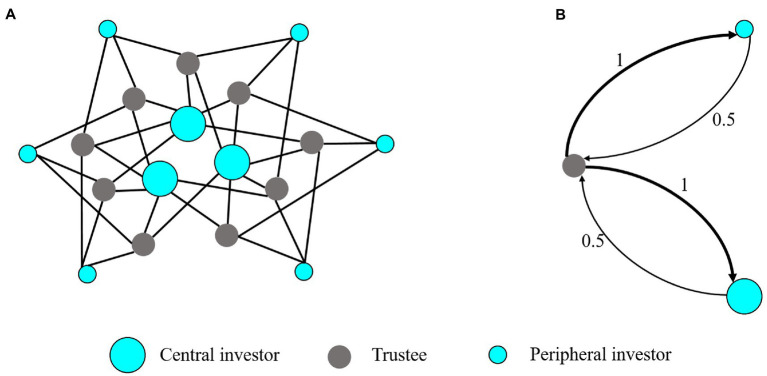
Network structure and mean-field approximation of the trust game. **(A)** The network structure of TG. The network has three central investors each connecting to six trustees; six peripheral investors, each connecting to three trustees, and nine trustees each connecting to four investors. **(B)** The mean-field approximation of the TG network. The simplified system consists of three (types of) nodes, a trustee, a peripheral investor, and a central investor, representing three typical players in the network. The afferent arrows represent interaction with the neighbors of nodes. The thickness of the arrow and the value on the arrow represents the connection link weight.

Due to the intensively studied effect of OT on trust, one may wonder whether trust (and altruism) increased by OT plays a role in fairness and cooperation in combination with inequity aversion and whether OT increases the trust of the whole network. In order to answer the questions, we analyze the trust game (TG) on the same heterogeneous network as that of UG (Figure 5A). Central and peripheral nodes are occupied by investors, and trustees have the same neighborhood size with four investor neighbors. Investors can choose a certain proportion of the initial endowment as an investment. Trustees receive the investment increased by a certain multiple and decide how much to return to their investors. Therefore, both investors and trustees can obtain benefits through trust and reciprocity. However, trustees can exploit trust not to return any resources. In analogy with the settings in UG, the actual payoff of subjects in a round is the mean payoff over the number of their neighbors.

### 4.2. Utility matrix

In general, we classify the behaviors of investors into two categories: invest (I) in trustees from the initial endowment or do not invest (NI). Analogously, we classify trustees into two categories: return (R) a part of the investment to investors or do not return (NR). In addition, we take the altruistic preference of trustees into account to better model their behaviors and assume that the increase in the utility of trustees is proportional to the return. In the experiment, there are two categories of investors, those occupying central nodes and those occupying peripheral nodes. Thus, we define two utility matrices between a central investor and a trustee, and between a peripheral investor and a trustee, respectively.

The utility matrix of a central investor and a trustee is shown in Table 9, where $T^{C}$ is the investment of a central investor with I strategy, r is the proportion of the investment that a trustee return to a central investor, g is the increase factor of investment ( g=3 in TG), $rgT^{C}$ is the return from a trustee, $gT^{C}\left(1-r\right)$ is the net gain of a trustee after returns $gT^{C}r$ , and the altruistic parameter λ ( λ>0 ) measures the willingness of return. In a similar manner, we define the utility matrix for a peripheral investor and a trustee (Table 9), where the superscript P denotes peripheral investors.

**Table 9:** Utility matrix between trustee and investor.

(a) Trustee vs. central investor	R	NR
I	$\left(1-T^{C}\right)+rgT^{C},gT^{C}\left(1-r\right)+\lambda T^{C}rg$	$1-T^{C},gT^{C}$
NI	1, 0	1, 0
(b) Trustee vs. peripheral investor	R	NR
I	$\left(1-T^{P}\right)+rgT^{P},gT^{P}\left(1-r\right)+\lambda T^{P}rg$	$1-T^{P},gT^{P}$
NI	1, 0	1, 0

We aim to investigate the immediate effect of OT on investment $T^{C}$ of central investors, and its possible indirect effect on investment $T^{P}$ of peripheral investors, and the altruistic parameter λ of a trustee. We also use mean-field approximation to simplify our analyses. Because of the normalization of payoffs over neighbors of every subject, the original network can be reduced to a three-node graph, as shown in Figure 5B. The simplified system consists of a trustee, a peripheral investor, and a central investor. The links in the original network are converted to the interaction weights in the simplified graph, where the principle of the approximation is similar to the case of network UG. Based on the approximation principle stemming from local interaction patterns, we can obtain the simplified network system, as shown in Figure 5B.

### 4.3. Replicator dynamics

Next, we formulate replicator dynamics equations of the simplified network system. We denote the probability of trustees using the R strategy by $\rho_{R}$ , the probability of central investors using the I strategy by $\rho_{I}^{C}$ , and the probability of peripheral investors using the I strategy by $\rho_{I}^{P}$ , respectively. According to the utility matrices, we can calculate the expected payoffs of subjects with different roles and strategies as follows:


(6) $$\begin{array}{c} \{\begin{array}{c} \begin{array}{c} E\left(R\right)=\frac{1}{2}\rho_{I}^{C}\left[gT^{C}\left(1-r\right)+\lambda T^{C}rg\right]+\frac{1}{2}\rho_{I}^{P}\left[gT^{P}\left(1-r\right)+\lambda T^{P}rg\right],\\ E\left(NR\right)=\frac{1}{2}\rho_{I}^{C}gT^{C}+\frac{1}{2}\rho_{I}^{P}gT^{P},\\ E^{C}\left(I\right)=\rho_{R}\left[\left(1-T^{C}\right)+rgT^{C}\right]+\left(1-\rho_{R}\right)\left(1-T^{C}\right),\\ E^{C}\left(NI\right)=1,\\ E^{P}\left(I\right)=\rho_{R}\left[\left(1-T^{P}\right)+rgT^{P}\right]+\left(1-\rho_{R}\right)\left(1-T^{P}\right),\\ E^{P}\left(NI\right)=1. \end{array} \end{array} \end{array}$$

where E(R) and E(NR) are the expected payoffs of trustees with R and NR strategies, $E^{C}\left(I\right)$ and $E^{C}\left(\mathrm{NI}\right)$ are the expected payoffs of central investors with I and NI strategies, and $E^{P}\left(I\right)$ and $E^{P}\left(\mathrm{NI}\right)$ are the expected payoffs of peripheral investors with I and NI strategies, respectively.

The three replicator dynamics equations for the three nodes in the simplified graph can be formulated as follows:


(7) $$\begin{array}{c} \{\begin{array}{c} \frac{d\rho_{R}}{dt}=\rho_{R}\left(E\left(R\right)-\bar{E}\right)=\rho_{R}\left(1-\rho_{R}\right)\left(E\left(R\right)-E\left(NR\right)\right),\\ \frac{d\rho_{I}^{C}}{dt}=\rho_{I}^{C}\left(E^{C}\left(I\right)-\bar{E^{C}}\right)=\rho_{I}^{C}\left(1-\rho_{I}^{C}\right)\left(E^{C}\left(I\right)-E^{C}\left(NI\right)\right),\\ \frac{d\rho_{I}^{P}}{dt}=\rho_{I}^{P}\left(E^{P}\left(I\right)-\bar{E^{P}}\right)=\rho_{I}^{P}\left(1-\rho_{I}^{P}\right)\left(E^{P}\left(I\right)-E^{P}\left(NI\right)\right), \end{array} \end{array}$$

where $\bar{E}$ , $\bar{E^{C}}$ , and $\bar{E^{P}}$ are the mean expected payoffs of trustees, central investors, and peripheral investors, respectively.

### 4.4. Stability analysis

There exist nine possible equilibrium points $\left(\rho_{R},\rho_{I}^{C},\rho_{I}^{P}\right)$ in the replicator dynamics equations, i.e., (0, 0, 0), (0, 0, 1), (0, 1, 0), (0, 1, 1), (1, 0, 0), (1, 0, 1), (1, 1, 0), (1, 1, 1), $\left(\frac{1}{rg},0,0\right)$ . We implement a stability analysis for each of the equilibrium points. For λ<1 , Eq. (7) has only one stable equilibrium, (0, 0, 0), but this equilibrium cannot explain all the experimental results. For λ>1 , Eq. (7) can have four possible stable states: (0, 0, 0), (1, 0, 1), (1, 1, 0), and (1, 1, 1). According to stability conditions, these stable states can be classified into three categories ( g=3 ) as follows:

- For r=0 , (0, 0, 0) is stable;
- For $r=\frac{1}{g}$ , (1, 0, 1) and (1, 1, 0) are stable;
- For $r>\frac{1}{g}$ , (1, 1, 1) is stable.

Subsequently, we analyze the stable points of each group of experiments to classify these groups into three categories. There are nine groups in the OT experiments and 10 groups in the PL experiments. The stable point and the classification of each group can be found in Supplementary Tables S2, S3.

The classification and stable point of experimental results demonstrate that our model is valid to characterize the evolutionary features of the trust experiment. In particular, we can see that the behavior r of the trustee is not affected by the investment T of investors or the altruistic parameter λ , and only the relation between r and g influences the category of experimental behaviors. In other words, OT that directly affects T and λ values plays a negligible role in the behavior of trustees, such that the dynamics of the experiment is not affected by OT as well. It is worth noting that OT, indeed, enhances the investment $T^{C}$ of central investors administrated OT by comparing with those of peripheral investors without inhaling OT (38). The results indicate that the effect of OT on enhancing the trust of investors is confined locally and cannot spread to other peripheral investors, due to the fact that the neighboring trustees of the central investors show no response to the generosity of the investors and shield the effect of OT. This finding is consistent with a pioneering experiment of one pair of investor and trustee, in which OT only affect the generosity of investors but is useless to trustees (58).

Taken together, locally administrated OT has no effect on the trust game experiments. This is mainly ascribed by the awarding from investors trigger by OT, which is not strong enough to motivate significant higher return of trustees. In contrast, in the UG and PD with costly punishment, the punishment stemming from inequality aversion triggered by locally administrated OT is effective to promote fairness and cooperation. In brief, group fairness and cooperative behavior can result from inequity aversion rather than trust.

## 5. Conclusion and discussion

Humans have a strong capacity to cooperate with genetically unrelated individuals. Yet because cooperation is exploitable by free-riding, when and how large-scale cooperation emerges and spreads through human social networks remains puzzling from both evolutionary and societal perspectives.

The effect of the heterogeneous network on group cooperation has always been a hot issue in related fields. Considering oxytocin is believed to be a neuropeptide with positive effects on prosocial behavior (e.g., positive effects on trust), the recent research conducted oxytocin-modulated network game experiments, including: the ultimatum game, the two-stage prisoner’s dilemma game (with the costly punishment stage), and the trust game in heterogeneous networks, respectively, and found that the administration of oxytocin (vs. matching placebo) to central individuals can increase the level of cooperation and fairness in the whole network significantly. Here, in order to further explore the intrinsic mechanism of this experimental phenomenon, we analyzed the evolution process of three game experiments in heterogeneous networks by constructing evolutionary game dynamics, respectively.

Based on the experimental data, the parameter estimation on the analytical results of the evolutionary game models shows that oxytocin can significantly enhance the prosocial preferences of the central subjects in all three games. In the UG and tPDG models, the altruistic punishment caused by inequality aversion is amplified and diffused through the heterogeneous network structure, thereby promoting cooperation and fairness in the overall network.

However, no cascading effects of oxytocin-induced prosocial behavior were observed in repeated rounds of TG experiments that did not involve inequality aversion (38). Oxytocin can significantly increase the investment (trust level) of investors, which is equivalent to a reward for the trustee (the incentive effect of reward is far weaker than punishment). However, the investor may lack a mechanism for punishment, and the trustee is not threatened with punishment and thus will not increase his/her return. In our model, we find that the trustee’s return ratio r is not affected by the investor’s investment T , which can effectively explain the experimental results. Therefore, we can conclude that the rewarding effect of trust is not sufficient to generate prosocial utility and that the costly punishment caused by inequality aversion is more effective in promoting the level of fairness and cooperation in the social network. These results confirm our hypothesis and may also explain existing network-free findings on punishment and reward (46, 59).

Our study opens an avenue to uncover general oxytocin-initiated mechanisms underpinning fairness and cooperation in human society through building evolutionary game models. Our evolutionary game model is a network variant of the replicator dynamics. Replicator dynamics have been widely used to study the evolution of cooperation and fairness in social networks (17, 60–62). One implicit assumption of the replicator dynamics is that imitation only occurs among individuals of the same type. While in the game experiments (38), subjects were also informed of the choices and payoffs of other types of subjects. Thus, they may not make decisions based on local imitation. However, it is worth noting that the goal of our study is not to exactly reproduce individual behaviors in the game experiments, but rather to show that the results observed in the experiments can be achieved and are evolutionarily stable in simple evolutionary game models.

In addition, our study provides an effective means to quantitively estimate the effect of oxytocin on inequality aversion and trust based on the experimental data. Thus, a possible direction for future research is to design experiments with different quantities of oxytocin, and we believe that our method can contribute to measuring how the quantity of oxytocin affects different social preferences. In short, our study and its future extension provide a new perspective for understanding the relationship between neuropeptides and prosocial behaviors.

## Data availability statement

The original contributions presented in the study are included in the article/Supplementary material, further inquiries can be directed to the corresponding authors.

## Author contributions

J-YL performed the analysis. J-YL, W-XW, and BZ wrote the first draft of the manuscript. All authors contributed to conception and design of the study, manuscript revision, and read and approved the submitted version.

## Funding

This study was supported by the National Natural Science Foundation of China (Nos. 11975049, 72131003, and 71922004), the Beijing Natural Science Foundation (No. Z220001), and the Collaborative Research Fund of the Chinese Institute for Brain Research, Beijing.

## Conflict of interest

The authors declare that the research was conducted in the absence of any commercial or financial relationships that could be construed as a potential conflict of interest.

## Publisher’s note

All claims expressed in this article are solely those of the authors and do not necessarily represent those of their affiliated organizations, or those of the publisher, the editors and the reviewers. Any product that may be evaluated in this article, or claim that may be made by its manufacturer, is not guaranteed or endorsed by the publisher.
