# Supplementary material for: Data-driven evolutionary game models for the spread of fairness and cooperation in heterogeneous networks
Source: Front Psychiatry. 2023 Apr 28;14:1131769. doi: 10.3389/fpsyt.2023.1131769 (PMC10204145; doi:10.3389/fpsyt.2023.1131769)
Supplement: Supplementary file 1 [file Data_Sheet_1.docx]

Supplementary Material

Evolutionary game analysis for the spread of fairness and cooperation in heterogeneous networks

Jing-Yi Li, Wen-Hao Wu, Ze-Zheng Li, Boyu Zhang ^*^

*** Correspondence:** Boyu Zhang: zhangby@bnu.edu.cn

# Supplementary Methods

## The ultimatum game on heterogeneous networks

**Replicator dynamics**

$$\begin{aligned} \left\{ \begin{aligned} &\frac{d\rho_{R}}{dt}=\rho_{R}\left( E\left( R \right)-\overline{E} \right)=\frac{1}{2}\rho_{R}\left( 1-\rho_{R} \right)\left( \rho_{C}^{C}+\rho_{C}^{P} \right)\left( \frac{1}{2}-s \right), \\ &\frac{d\rho_{C}^{C}}{dt}=\rho_{C}^{C}\left( E^{C}\left( C \right)-\bar{E^{C}} \right)=\rho_{C}^{C}\left( 1-\rho_{C}^{C} \right)\left\{ \rho_{R}\left[ s-\alpha_{1}\left( 1-2s \right) \right]+\frac{1}{2}\left( 1-\rho_{R} \right) \right\}, \\ &\frac{d\rho_{C}^{P}}{dt}=\rho_{C}^{P}\left( E^{P}\left( C \right)-\bar{E^{P}} \right)=\rho_{C}^{P}\left( 1-\rho_{C}^{P} \right)\left\{ \rho_{R}\left[ s-\alpha_{2}\left( 1-2s \right) \right]+\frac{1}{2}\left( 1-\rho_{R} \right) \right\}, \end{aligned} \right.\#\left( AUTONUM \backslash* Arabic \right) \end{aligned}$$

**Stability analysis**

We implement stability analysis for each of the equilibrium point. The general form of Jacobi matrix is

$$\begin{aligned} \left\{ \begin{aligned} \begin{aligned} &J_{11}=\frac{1}{2}\left( 1-2\rho_{R} \right)\left[ \rho_{C}^{C}\left( 0.5-s \right)+\rho_{C}^{P}\left( 0.5-s \right) \right], \\ &J_{12}=\frac{1}{2}\rho_{R}\left( 1-\rho_{R} \right)\left( 0.5-s \right), \\ &J_{13}=\frac{1}{2}\rho_{R}\left( 1-\rho_{R} \right)\left( 0.5-s \right), \\ &J_{21}=\rho_{C}^{C}\left( 1-\rho_{C}^{C} \right)\left\{ \left[ s-\alpha_{1}\left( 1-2s \right) \right]-0.5 \right\}, \\ &J_{22}=\left( 1-2\rho_{C}^{C} \right)\left\{ \rho_{R}\left[ s-\alpha_{1}\left( 1-2s \right) \right]+\left( 1-\rho_{R} \right)0.5 \right\}, \\ &J_{23}=0, \\ &J_{31}=\rho_{C}^{P}\left( 1-\rho_{C}^{P} \right)\left\{ \left[ s-\alpha_{2}\left( 1-2s \right) \right]-0.5 \right\}, \\ &J_{32}=0, \\ J_{33}=\left( 1-2\rho_{C}^{P} \right)\left\{ \rho_{R}\left[ s-\alpha_{2}\left( 1-2s \right) \right]+\left( 1-\rho_{R} \right)0.5 \right\}. \end{aligned} \#\#\# \end{aligned} \right.\#\left( AUTONUM \backslash* Arabic \right) \end{aligned}$$

For equilibrium points (0, 0, 0), (0, 0, 1), (0, 1, 0) and (0, 1, 1), there exist positive eigenvalues for $s<0.5$. Thus, they are unstable.

For point (1, 0, 0), we have

$$\begin{aligned} {J |}_{\left( 1,0,0 \right)}=\left( \begin{matrix} 0 & 0 & 0 \\ 0 & s-\alpha_{1}\left( 1-2s \right) & 0 \\ 0 & 0 & s-\alpha_{2}\left( 1-2s \right) \end{matrix} \right).\#\left( AUTONUM \backslash* Arabic \right) \end{aligned}$$

To guarantee that all eigenvalues are non-positive values, we have

$$\begin{aligned} \left\{ \begin{aligned} \begin{aligned} s<\alpha_{1}\left( 1-2s \right), \\ s<\alpha_{2}\left( 1-2s \right). \end{aligned}\#\#\#\#\#\#\#\#\#\#\# \end{aligned} \right.\#\left( AUTONUM \backslash* Arabic \right) \end{aligned}$$

For point (1, 0, 1), we have

$$\begin{aligned} {J |}_{\left( 1,0,1 \right)}=\left( \begin{matrix} -\frac{1}{2}\left( 0.5-s \right) & 0 & 0 \\ 0 & e-\alpha_{1}\left( 1-2s \right) & 0 \\ 0 & 0 & \alpha_{2}\left( 1-2s \right)-s \end{matrix} \right).\#\left( AUTONUM \backslash* Arabic \right) \end{aligned}$$

To guarantee that all eigenvalues are negative values, we have

$$\begin{aligned} \left\{ \begin{aligned} s<0.5, \\ \frac{\alpha_{2}}{1+2\alpha_{2}}<s<\frac{\alpha_{1}}{1+2\alpha_{1}}. \end{aligned} \right.\#\left( AUTONUM \backslash* Arabic \right) \end{aligned}$$

For point (1, 1, 0), we have

$$\begin{aligned} {J |}_{\left( 1,1,0 \right)}=\left( \begin{matrix} -\frac{1}{2}\left( 0.5-s \right) & 0 & 0 \\ 0 & \alpha_{1}\left( 1-2s \right)-s & 0 \\ 0 & 0 & s-\alpha_{2}\left( 1-2s \right) \end{matrix} \right).\#\left( AUTONUM \backslash* Arabic \right) \end{aligned}$$

To guarantee that all eigenvalues are negative values, we have

$$\begin{aligned} \left\{ \begin{aligned} s<0.5, \\ \frac{\alpha_{1}}{1+2\alpha_{1}}<s<\frac{\alpha_{2}}{1+2\alpha_{2}}. \end{aligned} \right.\#\left( AUTONUM \backslash* Arabic \right) \end{aligned}$$

For point (1, 1, 1), we have

$$\begin{aligned} {J |}_{\left( 1,1,1 \right)}=\left( \begin{matrix} s-0.5 & 0 & 0 \\ 0 & \alpha_{1}\left( 1-2s \right)-s & 0 \\ 0 & 0 & \alpha_{2}\left( 1-2s \right)-s \end{matrix} \right)\#\left( AUTONUM \backslash* Arabic \right) \end{aligned}$$

To guarantee that all eigenvalues are negative values, we have

$$\begin{aligned} \left\{ \begin{aligned} s<0.5, \\ \frac{\alpha_{1}}{1+2\alpha_{1}}<s, \\ \frac{\alpha_{2}}{1+2\alpha_{2}}<s. \end{aligned} \right.\#\left( AUTONUM \backslash* Arabic \right) \end{aligned}$$

Taken together, in a suitable range of offer $s$, (1,0,0), (1,0,1), (1,1,0), (1,1,1) are possibly stable.

Despite the stability of the four equilibrium points identified by stability analysis, the possibility of arriving at the points is different in the sense that their attraction is different. Specifically, the attraction of each equilibrium point can be quantified by the utility of proposers who dominate the resource allocation and are more self-interested in payoffs. If a point is associated with higher utilities, it will be of a higher probability to be the final stable state evolving from an arbitrary initial state. Let’s calculate the utility of proposers at the four equilibrium points. The general formula of the utility of proposers with R and F strategies is

$$\begin{aligned} E\left( R \right)=\frac{1}{2}\left( \rho_{C}^{C}+\rho_{C}^{P} \right)\left( 1-s \right),\#\left( AUTONUM \backslash* Arabic \right) \end{aligned}$$

and

$$\begin{aligned} E\left( F \right)=\frac{1}{4}\rho_{C}^{C}+\frac{1}{4}\rho_{C}^{P}.\#\left( AUTONUM \backslash* Arabic \right) \end{aligned}$$

By inserting the density of responders at the equilibrium points, we have

- For (1, 0, 0), $E\left( R \right)=0$ ，$E\left( F \right)=0$.
- For (1, 0, 1), $E\left( R \right)=\frac{1}{2}(1-s)$，$E\left( F \right)=\frac{1}{4}$.
- For (1, 1, 0), $E\left( R \right)=\frac{1}{2}(1-s)$，$E\left( F \right)=\frac{1}{4}$.
- For (1, 1, 1), $E\left( R \right)=1-s$，$E\left( F \right)=\frac{1}{2}$.

Note that at (1, 1, 1), both $E\left( R \right)$ and $E\left( F \right)$ reach the maximum value. Thus, (1, 1, 1) is of the highest likelihood to be the final stable state, i.e., proposers are rational and responders are cooperative.

## The two-stage Prisoner’s Dilemma game on heterogeneous networks

**Expected payoffs**

For central subjects, we have

$$\left\{ \begin{aligned} \begin{aligned} &E^{C}\left( C+N \right)=\frac{1}{4}\left\{ \hat{R}\left( \rho_{C+N}^{C}+\rho_{C+P}^{C} \right)+\left[ \hat{S}+\beta_{1}\left( \hat{R}-\hat{P} \right) \right]\left( 1-\rho_{C+N}^{C}-\rho_{C+P}^{C} \right) \right\} \\ +\frac{3}{4}\left\{ \hat{R}\left( \rho_{C+N}^{P}+\rho_{C+P}^{P} \right)+\left[ \hat{S}+\beta_{1}\left( \hat{R}-\hat{P} \right) \right]\left( 1-\rho_{C+N}^{P}-\rho_{C+P}^{P} \right) \right\}, \\ &E^{C}\left( C+P \right)=\frac{1}{4}\left\{ \hat{R}\left( \rho_{C+N}^{C}+\rho_{C+P}^{C} \right)+\left\{ \hat{S}-\hat{C}-\alpha_{1}\left\{ \left[ \left( \hat{T}-\hat{F} \right)-\left( \hat{S}-\hat{C} \right) \right]-\left( \hat{T}-\hat{S} \right) \right\} \right\}\left( 1-\rho_{C+N}^{C}-\rho_{C+P}^{C} \right) \right\} \\ +\frac{3}{4}\left\{ \hat{R}\left( \rho_{C+N}^{P}+\rho_{C+P}^{P} \right)+\left\{ \hat{S}-\hat{C}-\alpha_{1}\left\{ \left[ \left( \hat{T}-\hat{F} \right)-\left( \hat{S}-\hat{C} \right) \right]-\left( \hat{T}-\hat{S} \right) \right\} \right\}\left( 1-\rho_{C+N}^{P}-\rho_{C+P}^{P} \right) \right\}, \\ &E^{C}\left( D+N \right)=\frac{1}{4}\left\{ \hat{T}\rho_{C+N}^{C}+\left( \hat{T}-\hat{F} \right)\rho_{C+P}^{C}+\hat{P}\left( 1-\rho_{C+N}^{C}-\rho_{C+P}^{C} \right) \right\} \\ +\frac{3}{4}\left\{ \hat{T}\rho_{C+N}^{P}+\left( \hat{T}-\hat{F} \right)\rho_{C+P}^{P}+\hat{P}\left( 1-\rho_{C+N}^{P}-\rho_{C+P}^{P} \right) \right\}, \\ &\bar{E^{C}}=\rho_{C+N}^{C}E^{C}\left( C+N \right)+\rho_{C+P}^{C}E^{C}\left( C+P \right)+\left( 1-\rho_{C+N}^{C}-\rho_{C+P}^{C} \right)E^{C}\left( D+N \right). \end{aligned}\#( AUTONUM \backslash* Arabic ) \end{aligned} \right.$$

For peripheral subjects, we have

$$\left\{ \begin{aligned} \begin{aligned} &E^{P}\left( C+N \right)=\frac{1}{2}\left\{ \hat{R}\left( \rho_{C+N}^{C}+\rho_{C+P}^{C} \right)+\left[ \hat{S}+\beta_{2}\left( \hat{R}-\hat{P} \right) \right]\left( 1-\rho_{C+N}^{C}-\rho_{C+P}^{C} \right) \right\} \\ +\frac{1}{2}\left\{ \hat{R}\left( \rho_{C+N}^{P}+\rho_{C+P}^{P} \right)+\left[ \hat{S}+\beta_{2}\left( \hat{R}-\hat{P} \right) \right]\left( 1-\rho_{C+N}^{P}-\rho_{C+P}^{P} \right) \right\}, \\ &E^{P}\left( C+P \right)=\frac{1}{2}\left\{ \hat{R}\left( \rho_{C+N}^{C}+\rho_{C+P}^{C} \right)+\left\{ \hat{S}-\hat{C}-\alpha_{2}\left\{ \left[ \left( \hat{T}-\hat{F} \right)-\left( \hat{S}-\hat{C} \right) \right]-\left( \hat{T}-\hat{S} \right) \right\} \right\}\left( 1-\rho_{C+N}^{C}-\rho_{C+P}^{C} \right) \right\} \\ +\frac{1}{2}\left\{ \hat{R}\left( \rho_{C+N}^{P}+\rho_{C+P}^{P} \right)+\left\{ \hat{S}-\hat{C}-\alpha_{2}\left\{ \left[ \left( \hat{T}-\hat{F} \right)-\left( \hat{S}-\hat{C} \right) \right]-\left( \hat{T}-\hat{S} \right) \right\} \right\}\left( 1-\rho_{C+N}^{P}-\rho_{C+P}^{P} \right) \right\} \\ &E^{P}\left( D+N \right)=\frac{1}{2}\left\{ \hat{T}\rho_{C+N}^{C}+\left( \hat{T}-\hat{F} \right)\rho_{C+P}^{C}+\hat{P}\left( 1-\rho_{C+N}^{C}-\rho_{C+P}^{C} \right) \right\} \\ +\frac{1}{2}\left\{ \hat{T}\rho_{C+N}^{P}+\left( \hat{T}-\hat{F} \right)\rho_{C+P}^{P}+\hat{P}\left( 1-\rho_{C+N}^{P}-\rho_{C+P}^{P} \right) \right\}, \\ &\bar{E^{P}}=\rho_{C+N}^{P}E^{P}\left( C+N \right)+\rho_{C+P}^{P}E^{P}\left( C+P \right)+\left( 1-\rho_{C+N}^{P}-\rho_{C+P}^{P} \right)E^{P}\left( D+N \right). \end{aligned}\#( AUTONUM \backslash* Arabic ) \end{aligned} \right.$$

where $E^{C}\left( C+N \right)$, $E^{C}\left( C+P \right)$ and $E^{C}\left( D+N \right)$ are the expected payoffs of central node with C+N, C+P and D+N strategies, respectively, $E^{P}\left( C+N \right)$, $E^{P}\left( C+P \right)$ and $E^{P}\left( D+N \right)$ are the expected payoffs of peripheral node with C+N, C+P and D+N strategies, respectively, $\bar{E^{C}}$ and $\bar{E^{P}}$ are the mean expected payoffs of central node and peripheral node, respectively.

**Simulation**

We used $\left( \rho_{C+N}^{C}+\rho_{C+P}^{C} \right)*0.25+\left( \rho_{C+N}^{P}+\rho_{C+P}^{P} \right)*0.75$ as global cooperation rate and iterated from (0.5, 0.15, 0.15, 0.05) for 1000 generations in formula Eq.(5) in the main text and the simulation results of last 10 times are used as the final stable state (The variance of the last 10 times less than ${10}^{-10}$). In our experiment, $\alpha_{1}$*,* $\alpha_{2}$, $\beta_{1}$and $\beta_{2}$ in OT (PL) group are consistent with the estimated parameters.

## The trust game on heterogeneous networks

**Replicator dynamics**

$$\begin{aligned} \left\{ \begin{aligned} \begin{aligned} &\frac{d\rho_{R}}{dt}\boldsymbol{=}\rho_{R}\left( E\left( R \right)-\overline{E} \right)=\rho_{R}\left( 1-\rho_{R} \right)\left( E\left( R \right)-E\left( \mathrm{NR} \right) \right) \\ =\frac{1}{2}\rho_{R}\left( 1-\rho_{R} \right)\left[ \rho_{I}^{C}\left( \lambda rgT^{C}-rgT^{C} \right)+\rho_{I}^{P}\left( \lambda rgT^{P}-rgT^{P} \right) \right], \\ &\frac{d\rho_{I}^{C}}{dt}\boldsymbol{=}\rho_{I}^{C}\left( E^{C}\left( I \right)-\bar{E^{C}} \right)=\rho_{I}^{C}\left( 1-\rho_{I}^{C} \right)\left( E^{C}\left( I \right)-E^{C}\left( \mathrm{NI} \right) \right) \\ =\rho_{I}^{C}\left( 1-\rho_{I}^{C} \right)\left\{ \rho_{R}\left[ \left( 1-T^{C} \right)+rgT^{C} \right]+\left( 1-\rho_{R} \right)\left( 1-T^{C} \right)-1 \right\}, \\ &\frac{d\rho_{I}^{P}}{dt}\boldsymbol{=}\rho_{I}^{P}\left( E^{P}\left( I \right)-\bar{E^{P}} \right)=\rho_{I}^{P}\left( 1-\rho_{I}^{P} \right)\left( E^{P}\left( I \right)-E^{P}\left( \mathrm{NI} \right) \right) \\ =\rho_{I}^{P}\left( 1-\rho_{I}^{P} \right)\left\{ \rho_{R}\left[ \left( 1-T^{P} \right)+rgT^{P} \right]+\left( 1-\rho_{R} \right)\left( 1-T^{P} \right)-1 \right\}. \end{aligned}\# \end{aligned} \right.\#\left( AUTONUM \backslash* Arabic \right) \end{aligned}$$

**Stability analysis**

We implement stability analysis for each of the equilibrium point. The general form of Jacobi matrix is

$$\begin{aligned} \left\{ \begin{aligned} &J_{11}=\frac{1}{2}\left( 1-2\rho_{R} \right)\left[ \rho_{I}^{C}\left( \lambda rgT^{C}-rgT^{C} \right)+\rho_{I}^{P}\left( \lambda rgT^{P}-rgT^{P} \right) \right], \\ &J_{12}=\frac{1}{2}\rho_{R}\left( 1-\rho_{R} \right)\left( \lambda rgT^{C}-rgT^{C} \right), \\ &J_{13}=\frac{1}{2}\rho_{R}\left( 1-\rho_{R} \right)\left( \lambda rgT^{P}-rgT^{P} \right), \\ &J_{21}=\rho_{I}^{C}\left( 1-\rho_{I}^{C} \right)\left[ \left( 1-T^{C}+rgT^{C} \right)-\left( 1-T^{C} \right) \right], \\ &J_{22}=\left( 1-2\rho_{I}^{C} \right)\left\{ \rho_{R}\left[ \left( 1-T^{C} \right)+rgT^{C} \right]+\left( 1-\rho_{R} \right)\left( 1-T^{C} \right)-1 \right\}, \\ &J_{23}=0, \\ &J_{31}=\rho_{I}^{P}\left( 1-\rho_{I}^{P} \right)\left[ \left( 1-T^{P}+rgT^{P} \right)-\left( 1-T^{P} \right) \right], \\ &J_{32}=0, \\ J_{33}=\left( 1-2\rho_{I}^{P} \right)\left\{ \rho_{R}\left[ \left( 1-T^{P} \right)+rgT^{P} \right]+\left( 1-\rho_{R} \right)\left( 1-T^{P} \right)-1 \right\}. \end{aligned} \right.\#\left( AUTONUM \backslash* Arabic \right) \end{aligned}$$

The stability of equilibrium points are determined by the real part of the eigenvalues of the Jacobi matrix, as follows.

For equilibrium points (0, 0, 1), (0, 1, 0) and (0, 1, 1), there exist positive eigenvalues for $T^{C}>0$ and $T^{P}>0$, so they are unstable.

For point (0, 0, 0), we have

$$\begin{aligned} {J |}_{\left( 0,0,0 \right)}=\left( \begin{matrix} 0 & 0 & 0 \\ 0 & -T^{C} & 0 \\ 0 & 0 & -T^{P} \end{matrix} \right).\#\left( AUTONUM \backslash* Arabic \right) \end{aligned}$$

All eigenvalues are non-positive values, for $T^{C}>0, T^{P}>0$. Thus, (0, 0, 0) is stable.

For point (1, 0, 0), we have

$$\begin{aligned} {J |}_{\left( 1,0,0 \right)}=\left( \begin{matrix} 0 & 0 & 0 \\ 0 & rgT^{C}-T^{C} & 0 \\ 0 & 0 & rgT^{P}-T^{P} \end{matrix} \right).\#\left( AUTONUM \backslash* Arabic \right) \end{aligned}$$

To guarantee that all eigenvalues are non-positive values, we have $rg<1$.

For point (1, 0, 1), we have

$$\begin{aligned} {J |}_{\left( 1,0,1 \right)}=\left( \begin{matrix} -\frac{1}{2}\left( \lambda rgT^{P}-rgT^{P} \right) & 0 & 0 \\ 0 & rgT^{C}-T^{C} & 0 \\ 0 & 0 & T^{P}-rgT^{P} \end{matrix} \right).\#\left( AUTONUM \backslash* Arabic \right) \end{aligned}$$

To guarantee that all eigenvalues are non-positive values, we have $rg=1$.

For point (1, 1, 0), we have

$$\begin{aligned} {J |}_{\left( 1,1,0 \right)}=\left( \begin{matrix} -\frac{1}{2}\left( \lambda rgT^{C}-rgT^{C} \right) & 0 & 0 \\ 0 & T^{C}-rgT^{C} & 0 \\ 0 & 0 & rgT^{P}-T^{P} \end{matrix} \right).\#\left( AUTONUM \backslash* Arabic \right) \end{aligned}$$

To guarantee that all eigenvalues are non-positive values, we have $rg=1$.

For point (1, 1, 1), we have

$$\begin{aligned} {J |}_{\left( 1,1,1 \right)}=\left( \begin{matrix} -\frac{1}{2}\left[ \left( \lambda rgT^{C}-rgT^{C} \right)+\left( \lambda rgT^{P}-rgT^{P} \right) \right] & 0 & 0 \\ 0 & T^{C}-rgT^{C} & 0 \\ 0 & 0 & T^{P}-rgT^{P} \end{matrix} \right).\#\left( AUTONUM \backslash* Arabic \right) \end{aligned}$$

To guarantee that all eigenvalues are non-positive values, we have $rg>1$.

For point $\left( \frac{1}{rg},0,0 \right)$, we have

$$\begin{aligned} {J |}_{\left( \frac{1}{rg},0,0 \right)}=\left( \begin{matrix} 0 & \frac{1}{2rg}\left( 1-\frac{1}{rg} \right)\left( \lambda rgT^{C}-rgT^{C} \right) & \frac{1}{2rg}\left( 1-\frac{1}{rg} \right)\left( \lambda rgT^{P}-rgT^{P} \right) \\ 0 & 0 & 0 \\ 0 & 0 & 0 \end{matrix} \right).\#\left( AUTONUM \backslash* Arabic \right) \end{aligned}$$

All eigenvalues are zero.

Taken together, (0, 0, 0), (1, 0, 0), (1, 0, 1), (1, 1, 0), (1, 1, 1), $\left( \frac{1}{rg},0,0 \right)$ are possibly stable.

Further, we realize that some of the stable states identified by the stability analysis are not practical. Thus, we eliminate some unrealistic states that violate common sense. For example, state (1, 0, 0) cannot exist in the experiments in the sense that it is impossible for a trustee to return a positive amount even though investor invests nothing. Analogously, $\left( \frac{1}{rg},0,0 \right)$ is not possible either. Therefore, we eventually have four actually stable states: (0, 0, 0), (1, 0, 1), (1, 1, 0) and (1, 1, 1).

# Supplementary Tables

**Supplementary Table 1** The Jacob matrix and eigenvalues in tPDG.

| **Equilibrium point** | **Jacobi matrix** | **Eigenvalues** |
| --- | --- | --- |
| (0.1815,  0.1019,  0.2994,  0.0926) | $\left( \begin{aligned} -0.0366 0.0609 -0.1098 0.1828 \\ -0.0206 0.0342 -0.0617 0.1026 \\ -0.0973 0.1757 -0.0973 0.1757 \\ -0.0301 0.0543 -0.0301 0.0544 \end{aligned} \right)$ | -0.0412,  -0.0042,  0,  0 |
| (0.0917,  0.0583,  0.1535,  0.0514) | $\left( \begin{aligned} -0.0203 0.0382 -0.0608 0.1146 \\ -0.0129 0.0243 -0.0386 0.0729 \\ -0.0620 0.1211 -0.0620 0.1211 \\ -0.0208 0.0405 -0.0208 0.0406 \end{aligned} \right)$ | -0.0087+0.0132i, -0.0087-0.0132i,  0,  0 |

**Supplementary Table 2** The equilibrium points of each group identified by T-test in TG. r values of trustee in a group is obtained by averaging over the last 10 round of trustees. We implement T test to the r value by comparing with 0 and $\frac{1}{s}$. According to the p-value, classify stable points for each group.

|  | **group** | **stable point** |  | **group** | **stable point** |
| --- | --- | --- | --- | --- | --- |
| **OT** | Group 1 | (1, 0, 1) | **PL** | Group 1 | (0, 0, 0) |
|  | Group 2 | (1, 1, 0) |  | Group 2 | (0, 0, 0) |
|  | Group 3 | (1, 1, 0) |  | Group 3 | (0, 0, 0) |
|  | Group 4 | (0, 0, 0) |  | Group 4 | (1, 0, 1) |
|  | Group 5 | (0, 0, 0) |  | Group 5 | (1, 1, 0) |
|  | Group 6 | (0, 0, 0) |  | Group 6 | (0, 0, 0) |
|  | Group 7 | (1, 1, 0) |  | Group 7 | (0, 0, 0) |
|  | Group 8 | (0, 0, 0) |  | Group 8 | (1, 1, 0) |
|  | Group 9 | (1, 1, 1) |  | Group 9 | (1, 0, 1) |
|  |  |  |  | Group 10 | (1, 0, 1) |

**Supplementary Table 3** Number of groups in 3 kinds of stable points in TG experiment.

|  | **1^st^ type of stable points**  **(**$\mathbf{r=0}$**)** | **2^nd^ type of stable points**  **(**$\mathbf{r=}\frac{\boldsymbol{1}}{\boldsymbol{s}}$**)** | **3^rd^ type of stable points**  **(**$\mathbf{r>}\frac{\boldsymbol{1}}{\boldsymbol{s}}$**)** |
| --- | --- | --- | --- |
| **OT** | 4 | 4 | 1 |
| **PL** | 5 | 5 | 0 |
